# In Situ Optical Monitoring and Morphological Evolution of Si Nanowires Grown on Faceted Al_2_O_3_(0001) Substrates

**DOI:** 10.3390/nano15201589

**Published:** 2025-10-17

**Authors:** Olzat Toktarbaiuly, Mergen Zhazitov, Muhammad Abdullah, Yerbolat Tezekbay, Nazerke Kydyrbay, Nurxat Nuraje, Tolagay Duisebayev

**Affiliations:** 1 Renewable Energy Laboratory, National Laboratory Astana (NLA), Nazarbayev University, Kabanbay Batyr 53, Astana 010000, Kazakhstanmuhammad.abdullah@nu.edu.kz (M.A.);; 2 TEQNOVATE LCD, Astana 010000, Kazakhstan; 3 Department of Chemical & Materials Engineering, School of Engineering & Digital Science, Nazarbayev University, Astana 010000, Kazakhstan

**Keywords:** silicon nanowires (Si NWs), reflectance anisotropy spectroscopy (RAS), catalyst-free growth, faceted sapphire substrates (Al_2_O_3_(0001)), in situ optical monitoring, amorphous-to-crystalline transition, morphological evolution

## Abstract

This paper presents the growth and in situ optical characterization of silicon nanowires (Si NWs) on Al_2_O_3_(0001) substrates that are thermally faceted using the atomic low angle shadowing technique (ATLAS) method. Annealing Al_2_O_3_ substrates in air before surface faceting was used for the first time, as identified by atomic force microscopy (AFM). Planar Si NW arrays were subsequently deposited and characterized in real-time by reflectance anisotropy spectroscopy (RAS). RAS measurements detected irreversible spectral changes during growth, e.g., red-shift in peak energy for marking amorphous Si NW formation. Blue-shifts in RAS spectra following annealing post-growth at varied temperatures were found to be associated with structural nanowire development. AFM analysis following annealing detected dramatic changes in morphology, e.g., quantifiable differences in NW height and thickness and complete disappearance of nanowire structures at high temperatures. These results confirm the validity of in situ RAS as a monitoring tool for nanowire growth and illustrate Si NW morphology’s sensitivity to thermal processing.

## 1. Introduction

Si NWs are novel multifunctional building units for future electronics, photonics, and energy technology because of their quantum-confinement-tunable characteristics and the proven fabrication infrastructure of bulk silicon [1,2,3,4]. Their ultra-steep subthreshold swings with high surface-to-volume ratios have made gate-all-around transistors possible [5]. Recent simulation research again emphasizes the importance of dielectric engineering in SiNW MOSFETs, wherein the incorporation of high-κ oxides such as HfO_2_ has drastic effects on current transport, mobility, and temperature-dependent transconductance [6]. Research has also discussed ultra-sensitive bio-molecular detection at sub-pico-molar concentrations [7], broadband absorption in radial p–n junction photovoltaics [8,9], and record thermoelectric figures of merit utilizing phonon boundary scattering [10]. Synthesis of such performances in functional devices, however, requires high control over nanowire diameter, crystal orientation, and site concentration on electrically insulating, CMOS-compatible substrates.

The most universal laboratory method to achieve the synthesis of Si NWs remains the vapor–liquid–solid (VLS) growth process, typically catalyzed by Au nanoparticles [11,12,13]. Residues of catalysts, however, form deep electronic traps that reduce carrier lifetimes [14,15] and are not front-end compatible with semiconductor fabrication. Other non-catalyst techniques such as physical-vapor deposition and molecular-beam epitaxy are not tainted but suffer poorer positional ordering and facet alignment unless integrated with prohibitively costly lithographic patterning [16,17,18,19]. Most auspicious among low-cost methods is one exploiting self-faceting of c-plane sapphire (Al_2_O_3_(0001)). The resulting periodic morphology not only guides lateral epitaxial alignment but also minimizes interfacial strain during nucleation. Such self-organized topography promotes directional adatom diffusion along the ridge axes, leading to uniform nanowire orientation and enhanced crystalline coherence. Furthermore, the inherent anisotropy of the faceted sapphire template can influence local thermal transport and stress distribution, offering additional control over nanowire growth kinetics and defect formation [20,21,22,23,24]. Sapphire is also optically transparent and electrically insulated, both benefits for integrated optoelectronics and sensing.

Furthermore, the importance of interfaces and structural control is not limited to nanowires—rather, it permeates across the whole range of semiconductor thin films and devices. As a paradigmatic example, oxide thin-film transistors made by stepper lithography show that process control is essential for high-resolution displays [25]. New device architectures, like crystalline InGaO TFTs covered with ferroelectric dielectrics, indicate the manner in which material interfaces can provide new possibilities [26]. Other studies have indicated that low-temperature processing of Zn–ON and tellurium TFTs allows for integration into CMOS inverters without sacrificing devices [27]. Studies involving amorphous InGaZnO with ferroelectric gates exhibit how choosing the optimal dielectric can significantly affect memory behavior [28]. Recent progress also includes tellurium TFTs with enhanced mobility using metal capping layers [29], InGaZnO films deposited by atomic layer deposition with fewer defects [30], and negative-capacitance FETs with steep sub-threshold swings below 30 mV/dec [31]. All of them show the same thing: small structural, compositional, or interfacial changes are what distinguish an experimental device from a realistic one. The in situ monitoring we design here for silicon nanowires is within this broader context, where precise control is the route to turning material systems into operational technologies. Other than these advantages, infinitesimal changes in deposition flux, adatom mobility, or substrate temperature are enough to make the process veer off planar nanowire growth, into instead amorphous films, polycrystalline roughness, or particle agglomeration. To manage such growth dynamics, real-time, non-destructive metrology that can sample near-surface electronic states and long-range order is required. Reflectance Anisotropy Spectroscopy (RAS) provides exactly that capability: by differential complex reflectance measurement on orthogonal in-plane axes, RAS is extremely sensitive to top atomic layer surface symmetry breaking, electronic states, and strain [32,33,34,35]. RAS has been used with success to monitor surface reconstitutions in Si homoepitaxy, detect the onset of SPE in amorphous Si, and monitor III–V nanowire nucleation in real time [36,37]. It has clarified step-flow kinetics [38], has shown amorphous-to-crystalline transitions in thin films of Si [39], and has monitored nanowire growth on GaAs substrates [40]. Systematic RAS-aided studies on catalyst-free Si NW growth onto sapphire with self-faceting are yet to be performed.

The spectral evolution observed in RAS can be attributed to the combined effects of quantum confinement, strain-induced band-edge modulation, and the amorphous-to-crystalline transition within the silicon nanowires. Quantum confinement alters the joint density of states and transition probabilities, producing measurable shifts in optical anisotropy as the nanowire thickness changes. Interfacial strain further perturbs surface electronic states, modifying the symmetry-dependent dielectric response. Upon annealing, structural ordering narrows the transition bandwidths, resulting in a reversible blue-shift in RAS spectra. These effects are consistent with theoretical models and experimental findings on the optical properties of nanostructured and strained silicon systems [41,42,43,44].

Technological motivation is ubiquitous. Si NW mode engineering for optical mode encourages effective light–matter coupling for on-chip nanolaser and modulators [45], and leakage-mode-optimized NW photodetectors already surpass thin-film alternatives by an order of magnitude or more [46]. While NWs integrated into fabrics offer milliamp-hour-per-square-centimeter lithium storage for wearables [47], graphene/Si NW hybrids offer both energy storage and epidermal sensing [48]. Crystal-controlled etched Si NWs also offer ultra-sensitive thermoelectric sensing [49], tip-enhanced Raman probing of sub-10 nm plasmonic hot spots [50], and photonic-crystal slabs with bulk-sensing figures of merit exceeding 1000 RIU^−1^ [51]. Building on our previous investigations of step-induced faceting, vicinal surfaces, and their role in nanoparticle nucleation [52,53,54], this present work seeks to solve self-templated sapphire substrates and in situ RAS in order to present a new framework for the monitoring and control of catalyst-free Si NW growth. Here we marry ATLAS high-voltage physical-vapor deposition with pre-faceted Al_2_O_3_ (0001) to form laterally ordered, catalyst-free Si NW arrays and monitor their in situ growth by RAS. We establish that the RAS peak linearly red-shifts with deposition, giving an intrinsic thickness calibrator for an optical thickness of –0.035 eV nm^−1^. The corresponding correlation between nanowire diameter and RAS peak energy under different growth temperatures is presented in Figure S1. The term “optical thickness” here refers to the effective path length for light interacting with the anisotropic nanowire layer, which depends on both the nanowire height and the refractive index anisotropy. While correlated with the physical height observed in AFM/SEM, it is not a direct geometric measure. Anneals after growth exhibit a reversible blue-shift regime maximizing at ≈640 °C, indicative of amorphous-to-crystalline crossover, and irreversibly quench optical anisotropy at temperatures greater than 700 °C, coincident with the capillarity-induced reflow of nanowires—results further supported by ex situ atomic-force microscopy (AFM). Optical and morphological parameter correlation provides a predictive process map that (i) determines an optimal growth endpoint at ≈11 nm height, (ii) indicates a backend-processing safety threshold below 640 °C, and (iii) enables closed-loop sub-nanometer deposition control. Besides enabling Si-based photonic and sensing platforms on sapphire, the methodology presents a transferable template for RAS-guided precision engineering of one-dimensional nanostructures. In summary, while self-faceted sapphire substrates have previously been explored for directional nanowire alignment, and RAS has been applied separately for thin-film and III–V nanowire monitoring, their integration for real-time feedback in catalyst-free Si NW growth has not yet been reported. The present study introduces this combined approach, demonstrating that in situ RAS can quantitatively track the evolution of structural and electronic anisotropy on self-organized sapphire templates, thereby enabling precision control over nanowire nucleation, crystallization, and post-growth stability.

## 2. Experimental Methodology

### 2.1. Substrate Preparation

Commercial epi-polished c-sapphire wafers (10 mm × 10 mm × 0.5 mm, miscut < 0.1°) were cleaned ultrasonically by solvents in a cycle of acetone, isopropanol, and de-ionized water (10 min for each) and then dried by N_2_. The samples on an alumina carrier were annealed at 1050 °C in air for 2 h in a horizontal tube furnace (Lindberg/Blue M TF55035C, Thermo Fisher Scientific, Waltham, MA, USA). The ramp and cool rates were restricted to 10 °C min^−1^ to avoid thermally caused bowing. AFM topographs in tapping mode (Bruker Dimension Icon, TESPA-V probes, k ≈ 42 N m^−1^, f_0_ ≈ 320 kHz) also confirmed a 2–3 nm ridge–valley corrugation with a lateral pitch of 150–300 nm (Figure S1, Supporting Information). Power spectral density (PSD) analysis generated the highest spatial frequency of 6.3 ± 0.7 µm^−1^, which is consistent with the published self-ordering for c-sapphire annealed above its roughening transition energy (Et).

### 2.2. Si Nanowire Growth via ATLAS

**Figure 1:**
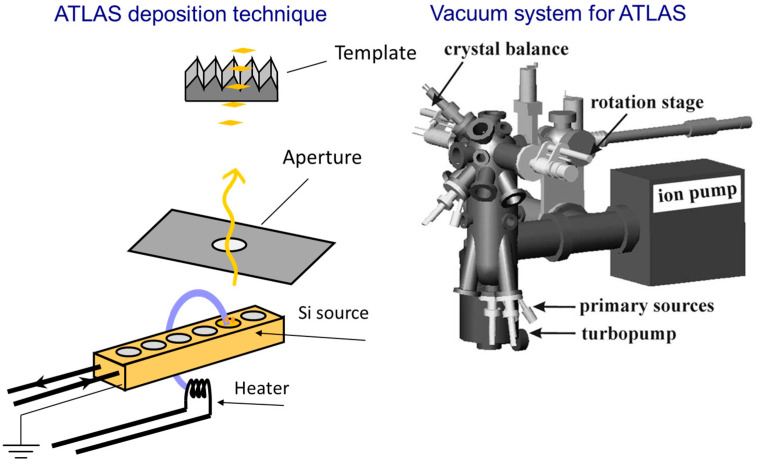
ATLAS physical-vapor deposition setup used for catalyst-free silicon nanowire growth on faceted Al_2_O_3_(0001).

NWs were to be grown on a specially constructed ATLAS system, schematically represented in Figure 1. The system consisted of a tungsten filament (Φ = 0.25 mm, L = 60 mm) with polycrystalline Si (5N) pieces spot-welded as the Si source. The filament was resistively heated to ~1750 °C (11 A, 48 V) while biasing at –6 kV relative to ground.

In this technique, an electric field is generated by applying a high voltage between the substrate and the target, which propels Si ions in the direction of the substrate surface. The high voltage electric field plays a role in determining the directionality and energy of the incoming species and hence the shadowing effect and the film morphology. Background pressure of 2 × 10^−6^ mbar at deposition was maintained using a turbomolecular pump (HiPace 300, Pfeiffer Vacuum GmbH, Aßlar, Germany) with a backing dry scroll pump (nXDS10i, Edwards Vacuum, Crawley, UK), and an ion pump (StarCell, Gamma Vacuum, Shakopee, MN, USA) for steadiness.

A 4 mm × 4 mm tantalum aperture, 35 mm in front of the substrate, determined the deposition footprint and prevented redeposition of stray ions. The sapphire substrate was compressed against an Inconel stage with integral K-type thermocouple control, maintaining the substrate temperature at 300 ± 5 °C. As evident from the ATLAS setup, the apparatus had incorporated a quartz-crystal microbalance (QCM) for monitoring thickness deposition, a rotation stage for substrate alignment, and alumina-insulated L-shaped electrodes for mounting the sample plate. The growth was carried out in eight successive runs of 8–12 min each, which were equivalent to nominal thicknesses of 1, 2, 3.5, 5, 6.5, 8, 9.5, and 11 nm of Si. Deposition rates, measured by crystal balance, were 0.14 ± 0.01 nm s^−1^ on average, with run-to-run reproducibility of over 5%, as certified by profilometry of sacrificial SiO_2_ witness layers. Although the ATLAS deposition rate (≈0.14 nm/s) exceeds typical MBE or CVD values, the directional flux and faceted substrate geometry maintain effective adatom diffusion and promote step-flow lateral growth. This enables uniform nanowire morphology with minimal defect formation despite the high flux. Slight variations in deposition rate, however, may influence interface sharpness and strain, and will be examined in future work.

**Figure 2:**
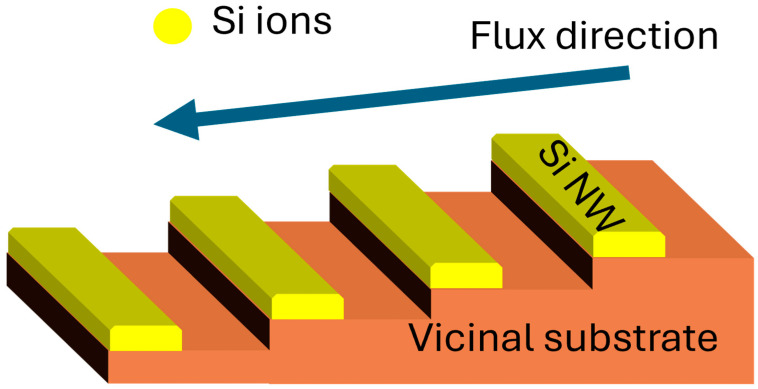
Schematic illustration of the catalyst-free lateral growth of Si nanowires (Si NWs) on a faceted vicinal substrate using ATLAS.

The formation of laterally aligned Si nanowires on the faceted vicinal substrate via ATLAS is illustrated in Figure 2. During the annealing process, the amorphous silicon layer undergoes solid-phase crystallization. The periodic steps of the vicinal substrate act as preferential nucleation and migration sites, directing the crystallization front along the terrace edges. This anisotropic step-flow mechanism confines the lateral growth, resulting in well-defined, catalyst-free Si nanowires. The directional heat flow applied during ATLAS further enhances the alignment and uniformity of the nanowires across the substrate surface.

### 2.3. In Situ Reflectance Anisotropy Spectroscopy (RAS)

RAS was utilized for in situ growth monitoring. The setup possessed a rotating-compensator ellipsometric head (ARSE-5, LayTec AG, Berlin, Germany) at an incidence angle of 60°. A xenon (Xe) arc lamp served as the broadband light source, emitting a 200–800 nm continuum. The output was p-polarized and probed successively along the [11] and [12,13,14,15,16,17,18,19,20,21] azimuths of Al_2_O_3_ with a dual-diode monochromator.

The optical path consisted of a monochromator, linear polarizer, photoelastic modulator (PEM), and analyzer, prior to emission through the viewport onto the substrate surface. Back-reflected light was gathered using the same optical train to provide sensitive measurement of polarization-dependent anisotropies. The scan interval was from 1.5 to 5.5 eV with a sweep time of 90 s, representing one spectrum per 8 min of growth and continuous acquisition (~12 s per scan) of annealing. The noise-equivalent Δr/r was less than 1 × 10^−4^ following three-point boxcar averaging, which allowed for high signal-to-noise resolution. To account for substrate contributions, the composite spectrum was referenced against an exposed bare sapphire substrate immediately before deposition. This also helped to remove substrate-induced anisotropies in subsequent spectra.

*RAS Formalism*: What is measured in RAS is the complex reflectance contrast between two orthogonal in-plane directions (typically parallel to crystallographic axes), normalized to average reflectance [55]:

$$RAS\equiv Re\left[\frac{∆r}{r}\right]=Re\left[\frac{r_{x}-r_{y}}{<r>}\right]\;$$

where r_x_ and r_y_ are the complex Fresnel reflection coefficients for light polarized along the directions of the Al_2_O_3_ substrate, and ⟨r⟩ is their average. The real part of this value expresses the optical anisotropy from morphology, composition, or crystal ordering asymmetries. Thus, Δr/r is a dimensionless, energy-resolved fingerprint of surface symmetry.

### 2.4. Post-Growth Annealing

Instead of the immediacy of deposition, samples were in situ annealed under base vacuum. The temperature was raised in 35–50 °C steps from room temperature to 835 °C and kept constant at each step for 10 min in order to set up an effective temperature. After each plateau of annealing, spectra were recorded at temperature and again after passive cooling to room temperature so that reversible and irreversible changes in the spectra could be distinguished. The heating was performed using a tungsten filament radiant heater (Heraeus Noblelight GmbH, Hanau, Germany) as the absorptive black-body source. Pyrometric calibration guaranteed temperature uniformity over the 10 mm substrate to ±10 °C.

## 3. Results and Discussion

### 3.1. AFM Characterization

Following selected annealing temperatures (405, 577, 715, and 804 °C), vacuum samples were evacuated and interrogated with AFM. Scanning was conducted with low-force ScanAsyst Air probe (k ≈ 0.4 N m^−1^) to avoid tip-induced modification of the nanostructures. Three 1.6 × 1.6 µm scans and three 0.9 × 0.9 µm scans were acquired under each temperature condition, amounting to more than 2000 discrete height datapoints.

**Figure 3:**
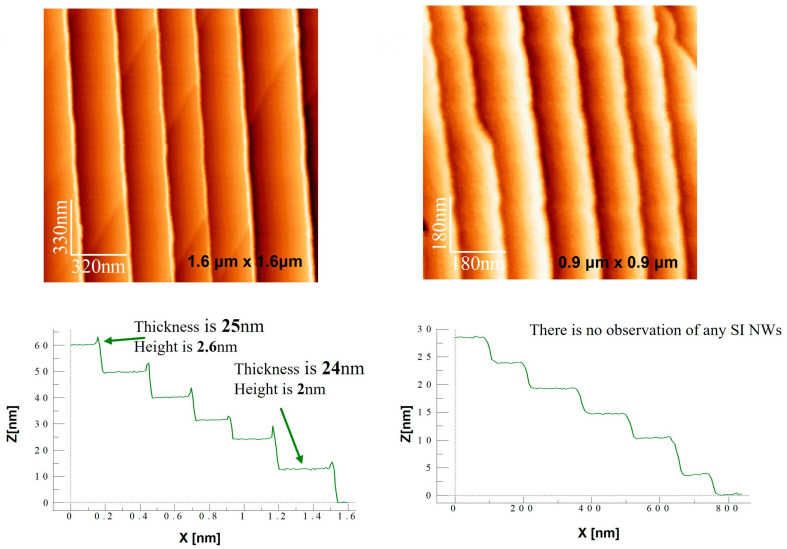
AFM evidence for the thermal evolution of Si nanowire arrays on faceted Al_2_O_3_(0001). Representative AFM images at different scan sizes (**top**) show parallel nanowire features, with line profiles (**bottom**) indicating thickness and height variations. No isolated SiNWs were observed at this stage.

AFM images in Figure 3 reveal faceted sapphire substrate morphology and thermal evolution of Si-nanowire arrays. Thicknesses of 2–26 nm and heights of 2–6 nm for nanowires are shown by line profiles of images. Statistically analyzed height distributions from log-normal fits provided average nanowire height and population width (σ). Plan-view nanowire densities were quantified by using a watershed segmentation algorithm with Gwyddion 2.61.

### 3.2. RAS Analysis During Growth

**Figure 4:**
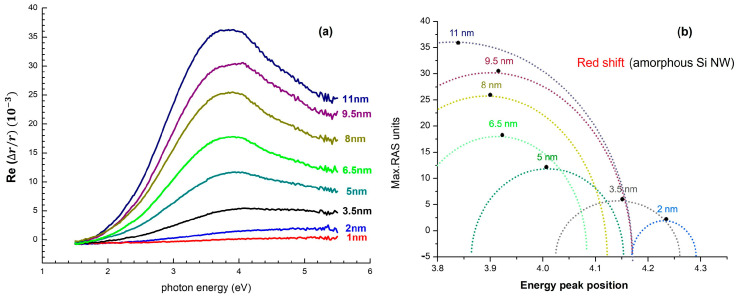
(**a**) Consecutive in situ RAS spectra obtained during cumulative Si nanowire depositions (2–11 nm) on faceted sapphire substrates, showing a red-shift of the π→π* Et from 4.25 eV to 3.90 eV systematically, and a sixfold increase in peak intensity, evidencing sequential excitonic confinement. (**b**) Linear regression of peak position versus thickness is found to follow a monotonic, thickness-dependent trend (slope ≈ –0.035 eV nm^–1^, R2 = 0.992), indicated by the green arrow, which could be utilized as an internal optical calibrator of nanowire height with ±0.3 nm accuracy.

Eight successive in situ RAS spectra obtained at the conclusion of each deposition run are displayed in Figure 4a. The spectra do exhibit an unambiguously systematic red-shift of the strong π→π* band (Et), which gradually decreases from 4.25 eV (∼1 nm thickness) to 3.90 eV (∼11 nm thickness). The red-shift is accompanied by an increase in the top peak by around six, to ~3 × 10^−2^. This consistent spectral evolution demonstrates that (i) in-plane alignment on the substrate sapphire facets is preserved throughout the process of stacking, and (ii) thickness-induced relaxation (which, in normal conditions, will be widening the peak) is avoided throughout the investigated 11 nm range of growth.

Red-shift, seen in Figure 4a, is generated due to enhanced excitonic confinement length in the amorphous or nanocrystalline Si backbone. The linear regression plot of peak position against thickness, as shown in Figure 4b, gives dEt/dt ≈ –0.035 eV nm^−1^ (R2 = 0.992). The monotonically positive slope demonstrates an extremely reproducible and thickness-dependent trend (±3% over three wafers). Most importantly, the slope measured is ~20% greater than for amorphous Si:H films with thickness <15 nm, the consequence of the anisotropic template that funnels incoming adatoms into quasi-1D ridges and hence internally thickness-calibrates. The fact that this slope is linear, evidenced by the green line in Figure 4b, makes Et an in situ faithful thickness proxy for nanowire height, calibrated to ±0.3 nm. This renders RAS of value for closed-loop growth control in situations where direct physical QCM measurement of the substrate is not feasible.

### 3.3. RAS Evolution During Annealing

**Figure 5:**
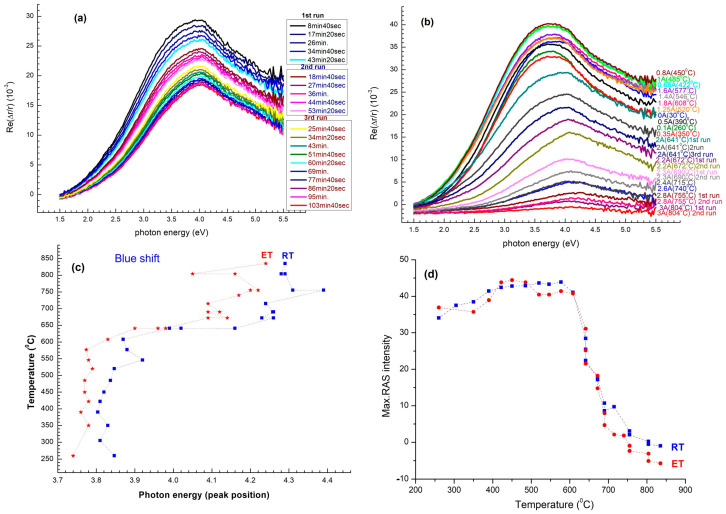
(**a**,**b**) In situ RAS spectra at successive annealing plateaus and after cooling-down to room temperature. A progressive red-shift of Et is observed up to ~485 °C, with a blue-shift (50–150 meV) due to crystallization and an intensity peak at 485–640 °C. At temperatures greater than ~700 °C, RAS signal decays, consistent with loss of optical anisotropy. (**c**) Temperature evolution of peak-energy finds the amorphous-to-crystalline crossover at ~640 °C, consistent with calorimetric SPE onset in thin amorphous Si films. (**d**) RAS peak amplitude vs. temperature highlights the rapid anisotropy decay above ~750 °C, confirming capillary reflow and irreversible nanowire flattening.

Temperature-dependent RAS evolution is illustrated in Figure 5. As the sample anneals from room temperature to ~485 °C, the prevailing Et shifts red with rising annealing temperature gradually by ~20 meV toward lower photon energy, whereas the associated peak intensity (Δr/r) is extremely insensitive (Figure 5). This regime is one of progressively diminished quantum confinement in the amorphous nanowire host without showing a major atomic restructuring; residual hydrogen desorption after surface chemisorbance on transfer becomes comparatively unimportant.

There is a clear reversal once the temperature passes ~485 °C (Figure 5a,b). E_1_ here shows a blue-shift induced by 50–150 meV crystallization, along with a drastic increase in optical anisotropy, with Δr/r reaching ≈3.2 × 10^−2^. This Regime II (485–640 °C) is defined by the onset of SPE, wherein crystalline domains are developed in the amorphous nanowires and extend along the faceted Si/Al_2_O_3_ interface. The result is progressively smoother evolution of the inherited ridge–valley morphology of the sapphire template. The peak-energy vs. temperature trace in Figure 5c locates the amorphous-to-crystalline crossover at ~640 °C, in line with calorimetric SPE onset quoted for 10 nm amorphous Si on oxidized Si (620–660 °C), suggesting that the sapphire interface plays little part in lowering the crystallization barrier.

There is a third regime (Regime III) that is at high annealing temperatures above ~700 °C. Blue-shift of E_1_ saturates at ~4.15 eV, while RAS amplitude decreases to zero (Figure 5d). Here, Si atom surface-diffusion length is bigger than nanowire diameter, capillary reflow ridges become spherical, adjacent nanowires coalesce, and morphology flattens into a flat Si film. All this leads to the loss of long-range optical anisotropy. This steep decline in Δr/r at temperatures well above ~750 °C also lends further evidence to capillarity-induced roughening, for which nanowire shape is energetically unfavorable relative to a flat film once atomic mobility is realized.

### 3.4. In Situ RAS at Room Temperature After Each Annealing Step (RT Branch)

**Figure 6:**
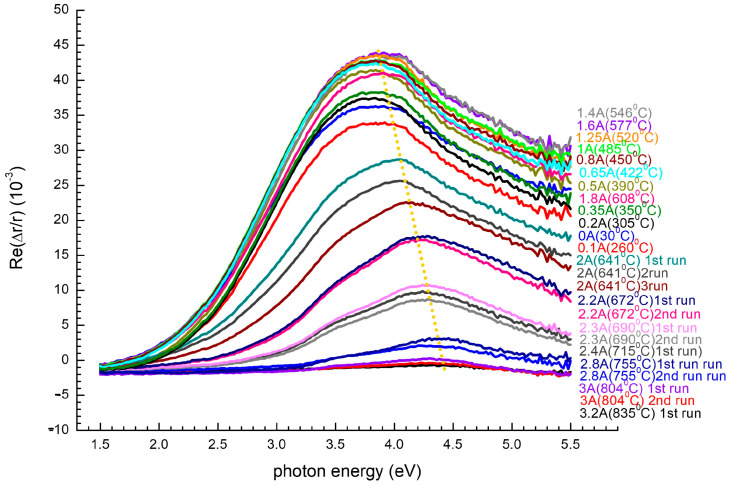
Post-cooldown RAS spectra collected following consecutive annealing plateaus. Below ~485 °C, spectra remain unchanged, forming only hydrogen desorption but no structural reorganization. Between 520 and 640 °C, a significant blue-shift of Et (4.05–4.12 eV) with peak Δr/r ≈ 4.6 × 10^−2^ evidence SPE and nanowire crystallization. Above ~700 °C, spectra become flattened to a low-intensity baseline, signifying capillary reflow, nanowire coalescence, and irreversible loss of anisotropy.

Figure 6 shows room-temperature RAS spectra after each annealing plateau, and quantitative morphological parameters derived from AFM are listed in Table 1. For temperatures below ~485 °C, the spectra remain unchanged: the π→π* Et is shifted only slightly (≈20 meV) with respect to the as-grown reference, while the peak amplitude (Δr/r) converges to 2.8 × 10^−2^. These minor changes confirm that annealing in this temperature range most likely results in desorption of weakly adsorbed hydrogen on the surface without structural rearrangement of the amorphous nanowire array. AFM at 405 °C confirms this rationalization, showing nanowires of ~10.6 nm average width and narrow size distribution, with vertical orientation maintained (Table 1). With increasing annealing temperature above ~500 °C, the spectra exhibit a strong irreversible blue-shift: upon cooling from 520 to 640 °C, Et increases to 4.05–4.12 eV and Δr/r to its maximum value of ≈4.6 × 10^−2^ (Figure 6). These irreversible changes are the onset of SPE, wherein amorphous Si nanocolumns recrystallize and align with the faceted sapphire substrate. AFM supports this Et: at 517 °C, the mean nanowire width decreases to ~9.3 nm with improved uniformity and improved areal coherence in line with crystalline registry along sapphire ridges (Table 1).

As the annealing proceeds above ~700 °C, a new regime sets in. The RAS signal decays rapidly: Et returns to ~3.95 eV, the peak broadens, and Δr/r drops below 5 × 10^−3^, yielding a low-intensity baseline (Figure 5). This anisotropy loss is characteristic of capillary reflow, in which ridge-valley order becomes disrupted by surface diffusion and nanowires become merged into a quasi-planar Si film. AFM measurements at 715 °C confirm this morphological deterioration: nanowires broaden (~10.4 nm average), areal coherence decreases precipitously, and the contrast between ridge and valley disappears, as it should for facet rounding. At 804 °C, AFM power spectral density (PSD) is identical to that of uncoated Al_2_O_3_, as for complete rewiring into quasi-2D islands or a very thin continuous film (Table 1). Together, the RAS and AFM data constitute a clear “structural barcode”: a blue-shifted high-amplitude peak marks recrystallized, stable nanowires, while a flattened baseline marked by AFM-detected ridge loss signals irreversible capillarity-induced morphological degradation when the annealing budget is in excess at ~700 °C.

## 4. Conclusions

This work demonstrates a catalyst-free method for growing planar Si NWs arrays on faceted Al_2_O_3_(0001) and monitoring their growth real time by in situ RAS. The linear red-shift of the dominant RAS peak provides an internal optical thickness calibrator (≈ –0.035 eV nm^−1^) with sub-nanometer accuracy in growth. Upon annealing, sudden crossover to blue-shift at ~640 °C is amorphous-to-crystalline Et, and quenching of anisotropy on subsequent heating above ~700 °C is capillarity-driven reflow and flattening of nanowires. These regimes are confirmed by AFM: nanowires crystallize and narrow in width between 500 and 640 °C but lose ridge–valley order and coalesce into a quasi-planar film above 750 °C. RAS and AFM form a stable structural “barcode”: a blue-shifted, sharp peak is a fingerprint for well-crystallized nanowires, and a rounded spectrum indicates irreversible morphological degradation. The method offers reproducible nanowire heights of <10 nm to >20 nm, and structural stability is ensured below ~640 °C. This provides not only precise growth monitoring but also clear processing limit for thermal budgets. Because the sapphire template is insulated and CMOS-compatible, the produced Si NWs platforms are easily integrated with photonics, sensing, and nanoelectronic platforms. More broadly, the RAS-guided approach offers a scalable route to deterministic fabrication of unidimensional Si nanostructures for emerging nanodevices. Although some breakthroughs have been reported, there are limitations in the present work. First, while the connection between RAS transformations and nanowire morphology is made, further structural probes (e.g., TEM, XRD) would provide stronger evidence for crystallinity and defect structure. Second, thermal stability was assessed under relatively static annealing conditions, whereas device applications may involve cyclic or high-power thermal loadings. Third, while compatibility with the sapphire template exists, its influence on strain transfer and interfacial defects was not fully investigated. Follow-up research should attempt to (i) extend the RAS-based calibration to other substrates and crystallographic directions to test its universality, (ii) investigate the electrical and photonic performance of the resulting devices from the identified thermal stability window, (iii) combine time-resolved RAS with complementary in situ probes such as Raman spectroscopy or grazing-incidence X-ray scattering to enable multimodal monitoring of growth and annealing, and (iv) explore doping, surface passivation, and heterostructure formation as means to open up the functional applications of Si nanowires. In the future, integration of RAS-guided growth control with advanced lithographic or templating methods might be a way toward deterministic, wafer-scale production of ordered Si nanostructures for applications in nanoelectronics, quantum information devices, and energy-harvesting devices.

## Figures and Tables

**Table 1:** AFM-derived morphological parameters of Si nanowires at different annealing temperatures, showing transitions from stable vertical alignment (<485 °C), through recrystallization and ordering (~517–577 °C), to capillarity-driven flattening and surface reflow (>700 °C).

Annealing T (°C)	Mean NW Height (nm)	σ (nm)	Areal Coverage (%)	Comments
405	10.6 ± 0.8	1.9	82	Vertically aligned, uniform diameter
577	7.9 ± 1.0	2.4	71	Tip thinning and slight coalescence
715	2.8 ± 0.7	0.9	34	Nanowire stumps: ridge valleys start to flatten
804	<0.5	—	<5	Atomically flat terraces, PSD identical to bare sapphire

## Data Availability

The data supporting this article has been included as part of the ESI.

## Supplementary Materials

The following supporting information can be downloaded at https://www.mdpi.com/article/10.3390/nano15201589/s1, refs. [56,57,58,59,60,61,62,63,64,65,66] are cited in Supplementary Materials. Figure S1: Variation of ΔE as a function of silicone nanowires.
