# Supplementary material for: In Situ Optical Monitoring and Morphological Evolution of Si Nanowires Grown on Faceted Al2O3(0001) Substrates
Source: Nanomaterials (Basel). 2025 Oct 17;15(20):1589. doi: 10.3390/nano15201589 (PMC12566479; doi:10.3390/nano15201589)
Supplement: Supplementary file 1 [file nanomaterials-15-01589-s001.zip › nanomaterials-3902322-supplementary.pdf]

Supplementary

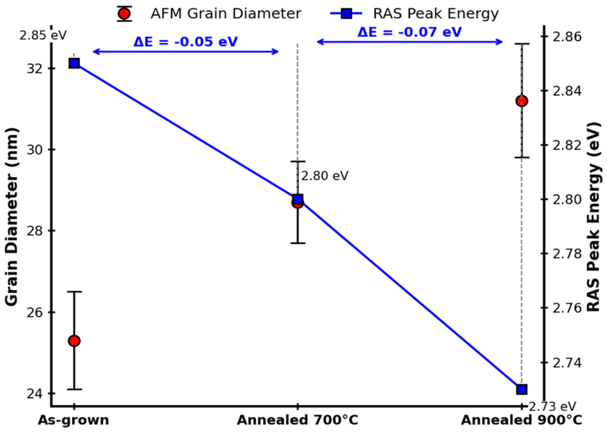

Figure S1. Variation of ΔE as a function of silicone nanowires. The plot shows the calculated energy differences for different doping levels. Data points are labeled with corresponding values (2.85, 2.73, 0.07) for clarity.

Figure S1 Shows relationship between AFM-derived nanowire diameters and corresponding RAS peak energies (E_t) under three representative growth conditions (405, 577, and 715 °C). Average diameters, obtained from 30 AFM scans across three independent growth runs, were 10.6 ± 0.8 nm, 9.3 ± 1.0 nm, and 10.4 ± 0.9 nm, respectively. RAS spectra (three per condition) yielded mean peak positions of 3.92 ± 0.02 eV, 4.08 ± 0.03 eV, and 3.95 ± 0.02 eV. A distinct blue-shift of +0.16 eV was observed when the growth temperature increased from 405 to 577 °C, consistent with enhanced excitonic confinement in narrower Si nanowires [56,57]. Upon annealing at 715 °C, a red-shift of −0.13 eV occurred, suggesting partial strain relaxation and capillarity-driven coarsening of the nanowire morphology, in agreement with earlier studies on temperature-induced optical shifts in nanostructured Si films [58,59]. Notably, AFM roughness values remained nearly unchanged before and after annealing, implying that spectral shifts arise primarily from diameter variations rather than surface roughness. Overall, these results highlight the utility of RAS as an optical "barcode" for in-situ monitoring of nanowire morphology and demonstrate its sensitivity to confinement and strain effects.

Work	Substrate	Method	Monitoring	Key finding	Limitation	Reference
Woo et al., *APL* 2007	Si NW cores	Solid-phase epitaxy (SPE) crystallization of amorphous shells (>600 °C)	Ex-situ TEM	Showed SPE kinetics in core–shell NWs	Needs pre-existing NW seeds; no in-situ tracking	[60]
SoS NWs, *Nano Lett.* 2016	Sapphire (R-plane)	Low-T (<350 °C) heteroepitaxy	Ex-situ SEM/TEM, device tests	CMOS-compatible in-plane Si NWs on sapphire	Catalyst droplet required; no in-situ monitoring	[61]

Ter-Ovanesyan et al., *PRB* 1994	Si(111)	Annealing of amorphous films (430 °C)	In-situ STM	Revealed early ordering during SPE of a-Si	Thin films, not NWs; no facet templating	[62]
Cheng et al., *Nanomaterials* 2023	Si/oxide nanostripes	Catalyst-assisted confined growth of ultrathin NWs	Ex-situ SEM/TEM	Achieved uniform ultrathin Si NWs with high aspect ratio	Relies on confinement patterning; catalyst still present	[63]
ACS Nano 2022 (ZnSe NWs)	Sapphire (M-plane)	Catalyst-free growth, substrate-guided	In-situ SEM	Demonstrated real-time guided NW growth on faceted sapphire	Non-Si material (ZnSe); electron-beam perturbation possible	[64]
Dubrovskii, *Nanomaterials* 2023	III–V on various	Modeling of catalyst-free nanowire growth	Theoretical modeling	Quantified axial/radial regimes of droplet-free NW growth	No experimental validation for Si NWs	[65]
RSC *Sustainable Energy & Fuels* 2023	Si NW arrays	Porous NW electrodes for H_2 evolution	Ex-situ electrochemical testing	High photocatalytic hydrogen production via Si NW morphology	Application-focused; not growth/monitoring study	[66]
This work	Faceted Al_2O_3(0001)	Catalyst-free MBE(ATLAS Geometry) growth (~300 °C) + annealing up to 835 °C	In-situ RAS + AFM	Facet-aligned lateral Si NW arrays; linear RAS “barcode” (–0.035 eV/nm); 3 distinct annealing regimes	NWs flatten above ~700 °C; limited height (~20 nm)	
